# Comparison of viremia of type II porcine reproductive and respiratory syndrome virus in naturally infected pigs by zip nucleic acid probe-based real-time PCR

**DOI:** 10.1186/1746-6148-9-181

**Published:** 2013-09-12

**Authors:** Chao-Nan Lin, Wei-Hao Lin, Li-Ning Hung, Sheng-Yuan Wang, Ming-Tang Chiou

**Affiliations:** 1 Department of Veterinary Medicine, College of Veterinary Medicine, National Pingtung University of Science and Technology, Pingtung 91201, Taiwan, Republic of China; 2 Veterinary Hospital, College of Veterinary Medicine, National Pingtung University of Science and Technology, Pingtung 91201, Taiwan, Republic of China

**Keywords:** Porcine reproductive and respiratory syndrome virus, Viral load, Zip nucleic acid, Real-time PCR

## Abstract

**Background:**

Porcine reproductive and respiratory syndrome virus (PRRSV) is a RNA virus with high genetic variation. This virus causes significant economic losses in most pig-producing countries. The clinical presentation of PRRSV ranges from asymptomatic to devastating. In this study, we developed a sensitive and specific zip nucleic acid probe-based real-time PCR assay to evaluate the viremia of natural PRRSV-infected pigs in Taiwan. Serum samples were collected from 577 pigs aged 5–12 weeks. These include 444 clinically healthy pigs and 133 symptomatic pigs were confirmed to have porcine respiratory disease complex (PRDC).

**Results:**

Viremia was quantified in 79 of the 444 (17.8%) clinically healthy pigs and in 112 of the 133 (84.2%) PRDC cases. Viremias were significantly more common in pigs with PRDC compared with the clinically healthy pigs (*P* <0.0001). These results suggest that a high viral load is a major feature of PRRSV-affected pigs.

**Conclusions:**

ZNA probe-based real-time PCR can be a useful tool to diagnose symptomatic and asymptomatic PRRSV-infected pigs. The presence of this marker in a sample of animals with high PRRSV loads (>10^4.2^ PRRSV genomes/μl of serum) seems to indicate that it correlates with the presence of PRDC in pigs.

## Background

Porcine reproductive and respiratory syndrome (PRRS) causes significant economic losses in most pig-producing countries [1]. The causative agent, the PRRS virus (PRRSV), was identified in the early 1990s [1]. PRRSV is an enveloped, positive-strand RNA virus with a genome of approximately 15 kb, and it belongs to family *Arteriviridae* and order *Nidovirales*[1]. A remarkable amount of genetic variation has been observed among the PRRSVs isolates worldwide, particularly in Nsp2 [2-5], ORF5 [2-8] and the nucleocapsid (the ORF 7 product) [3,5,8,9]. The genetic characteristics of the PRRSV strains clearly indicate the existence of two major genotypes, the European type (EU genotype, type 1) and the North American type (NA genotype, type 2) [1].

During PRRSV infection, clinical disease is detectable in all of ages of pigs but is usually observed in nursery-grown pigs [1]. The clinical presentation of PRRSV can range from asymptomatic to devastating, with symptoms such as listlessness, emaciation, hyperpnea, dyspnea, chemosis, abortion, stillbirth and a reduction in semen quality [1]. However, infection with PRRSV predominantly exists at a subclinical level, participating as a cofactor in porcine respiratory disease complex (PRDC) and porcine circovirus associated disease (PCVAD) [10]. PRRSV can be detected using molecular methods from nasal fluid, salivary, serum and tonsil specimens from naturally infected pigs [11]. However, because PRRSV is common within the swine population, no quantitative real-time PCR assays have been described using the serum samples of both symptomatic and asymptomatic PRRSV-infected pigs.

The diagnosis of PRRSV infection has relied on probe-based real-time PCR [12-21], SYBR Green-based PCR [11,22-24], RT-PCR [1], virus isolation [1], immunohistochemistry [1] and serological methods [1]. Zip nucleic acids (ZNA) are oligonucleotide-oligocation conjugates with multiple cationic spermine moieties attached to the nucleic acid oligomer [25]. The melting temperature of a hybridized ZNA is easily predictable and increases linearly with the length of the oligocation [25]. ZNA were shown to enable specific and sensitive reactions when used as a primer for PCR and reverse transcription [26]. The present study describes a sensitive method for detecting type 2 PRRSV using real-time fluorescent quantitative PCR with ZNA probes.

## Results

### ZNA probe-based real-time PCR amplification and the limit of detection

**Figure 1:**
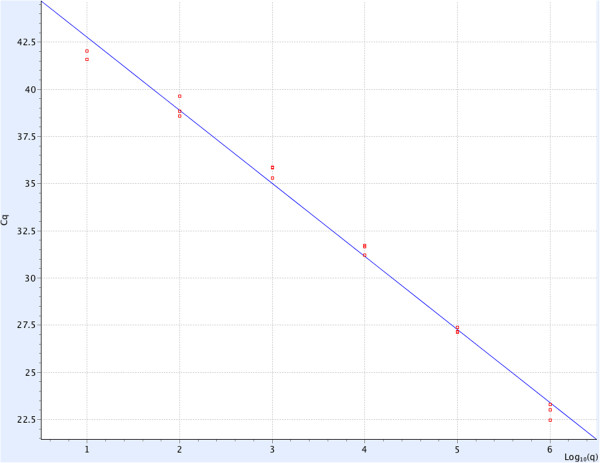
**ZNA probe-based real-time PCR assay showing results obtained with serial dilutions of the PRRSV plasmid.** Regression lines between the cycle threshold values plotted against the logarithm of the input copy number of the standard plasmid DNA. The dynamic range of the real-time PCR assay spanned 6 log units with a slope of −3.91 and an *R*^*2*^ value of 0.996.

Tenfold serial plasmid dilutions (10^1^ to 10^6^ copies/μl) were tested and used to construct the standard curve by plotting the logarithm of the plasmid copy number against the measured quantification cycles (Cq) values. The generated standard curve covered a linear range of six orders of magnitude of the standard plasmid DNA. The linear correlation (*R*^*2*^) between the Cq and the logarithm of the plasmid copy number was 0.996 (slope = −3.91) (Figure 1). To assess the limit of detection of the assay, 10^0^ to 10^6^ copies/μl of standard plasmid DNA per reaction were tested in 10 replicates. At more than 100 copies, 100% of the replicates were positive, and at 10 copies, 6 (60%) of the replicates were positive (Table 1).

**Table 1:** Efficiency of the PRRSV ZNA probe-based real-time PCR assay

**Estimated PRRSV plasmid DNA copy no.**	**N**	**Mean Cq ± SD**
10^1^	6/10^a^	41.69 ± 0.42
10^2^	10/10	39.19 ± 0.99
10^3^	10/10	35.36 ± 0.55
10^4^	10/10	31.28 ± 0.43
10^5^	10/10	27.55 ± 0.55
10^6^	10/10	22.63 ± 0.54

^a^Number positive/number tested.

### Reproducibility and specificity of the ZNA probe-based real-time PCR

The coefficient of variation of the mean Cq values in the within-run and between-run for standard plasmid DNA precision experiments ranged from 0.52 to 1.87% and from 0.85 to 1.98%, respectively (Table 2). The CV values in the within-run and between-run for clinical specimens that spanned the whole ranged from 0.43 to 4.70% and from 2.11 to 4.45%, respectively (Table 3). We analyzed other swine viruses to test the specificity of the ZNA probe-based real-time PCR. No specific amplifications were detected for any of these samples (data not shown).

**Table 2:** Reproducibility of the PRRSV ZNA probe-based real-time PCR assay

**Concentration of the standard plasmid (copies/μl)**	**Intra-assay variability**		**Inter-assay variability**	
	**Mean Cq ± SD**	**CV%**	**Mean Cq ± SD**	**CV%**
10^6^	22.89 ± 0.43	1.87	23.95 ± 0.47	1.98
10^5^	27.18 ± 0.14	0.52	27.75 ± 0.24	0.85
10^4^	31.50 ± 0.28	0.88	32.06 ± 0.31	0.98
10^3^	35.63 ± 0.31	0.87	36.34 ± 0.35	0.96
10^2^	38.99 ± 0.56	1.43	40.50 ± 0.85	1.26

**Table 3:** Results of the intra-assay and inter-assay testing for clinical specimens

**Pig no.**	**Intra-assay variability**		**Inter-assay variability**	
	**Mean Cq ± SD**^**a**^	**CV%**	**Mean Cq ± SD**	**CV%**
1	2.09 ± 0.06	2.68	2.13 ± 0.05	2.11
2	2.98 ± 0.14	4.70	3.04 ± 0.14	4.45
3	4.85 ± 0.02	0.43	4.85 ± 0.16	3.39
4	6.01 ± 0.09	1.45	6.07 ± 0.17	2.72
5	2.75 ± 0.13	4.60	2.76 ± 0.10	3.51

^a^Log10 copy number per microliter of PRRSV.

### Detection of the viral load in serum samples from in the PRRSV naturally infected pigs

**Figure 2:**
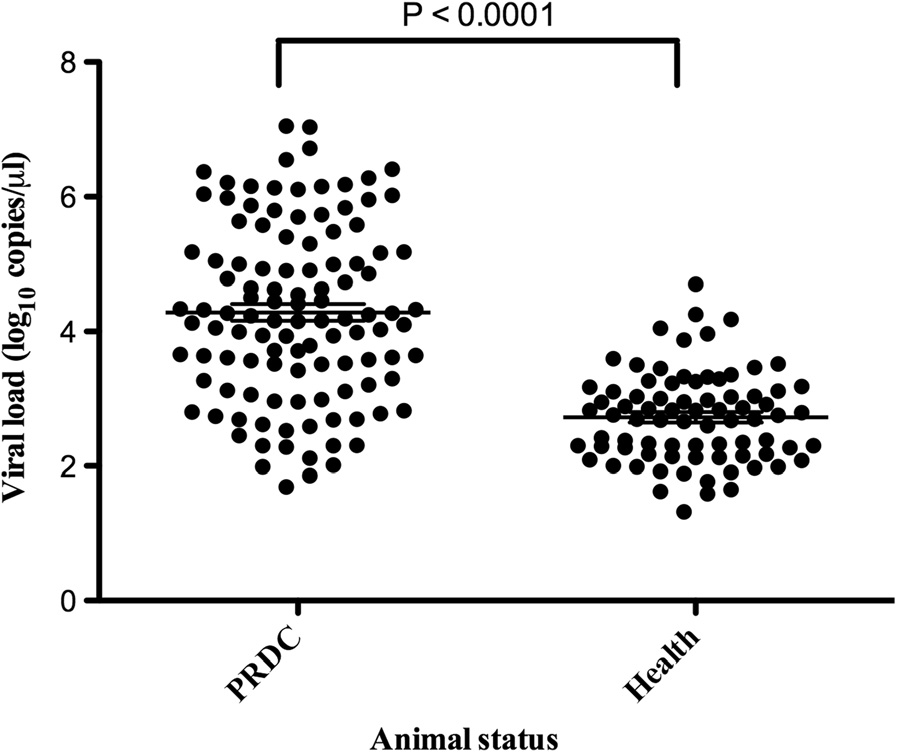
**Absolute copy number per microliter of PRRSV in serum from PRDC and clinically asymptomatic pigs.** The serum of PRDC (n = 112) and asymptomatic pigs (n = 79) was analyzed using ZNA probe-based real-time PCR. The mean percentages (long horizontal lines) of the PRRSV load in the PRDC and asymptomatic pigs were compared. Error bars show SD. The PRRSV load was significantly higher (P < 0.0001) in the pigs with PRDC compared with the asymptomatic pigs, as assessed by unpaired, 2-tailed Student’s *t*-tests.

The assay was tested on serum from both healthy and PRDC pigs. As determined by ZNA probe-based real-time PCR, 112 (84.2%) of the 133 PRDC pigs and 79 (17.8%) of the 444 asymptomatic pigs were positive (Table 4). Using a chi-square test, the correlation between PRRSV and PRDC was calculated. Among the 577 pigs that were analyzed, a positive result for PRRSV appeared to be highly significantly correlated with the presence of PRDC (*P* <0.0001). In addition, the PRRSV load was significantly higher (*P* <0.0001) in the PRDC pigs (ranging from 1.69 to 7.05 log_10_ PRRSV genome/μl, median 4.21 log_10_) compared to that in the asymptomatic pigs (ranging from 1.32 to 4.70 log_10_ PRRSV genome/μl, median 2.75 log_10_) (Figure 2) (Table 4). These data indicated that the levels of viral load correlated with the severity of the clinical presentation of the PRRSV-infected pigs.

**Table 4:** Number and descriptions of the PRRSV load results in serum collected from 2012 to 2013

	**Animal status**		
	**PRDC**	**Asymptomatic**	***P *value**
Number of tested pigs	133	444	
Number of positive pigs (%)	112 (84.2)	79 (17.8)	<0.0001
Mean ± SD^a^	4.28 ± 1.31	2.72 ± 0.67	<0.0001
Median^a^	4.21	2.75	
Range^a^	1.69 to 7.05	1.32 to 4.7	

^a^Log 10 Copies number per microliter in serum.

## Discussion

The molecular diagnosis of PRRSV infection has relied on probe-based real-time PCR [12-21] and SYBR Green-based PCR [11,22-24]. This study first describes a new ZNA probe-based real-time PCR for PRRSV and evaluates this method as a diagnostic tool for PRRSV infection. This assay was sensitive, specific and reliable for the amplification of PRRSV cDNA, with a reproducible limit of detection of approximately 100 copies of target DNA per reaction. Both the intra- and inter-assay CVs for standard plasmid DNA and clinical specimens were satisfactorily low.

A novel type of modified oligonucleotides known as ZNA was recently developed as a method for the clinical diagnosis of pathogens, such as the hepatitis B virus [27]. ZNAs are able to discriminate between complementary sequence that are a perfect match and those that are mismatched by a single base pair [25]. ZNAs are particularly efficient at low magnesium concentrations, low primer concentrations and high annealing temperatures [25]. ZNA probes provide broad flexibility with respect to experimental design and represent an effective alternative to minor groove binder-DNA and locked nucleic acid probes [26].

The viral load is an indicator of active infection, virus-host interaction and disease progression [28]. The asymptomatic pigs are believed to serve as important reservoirs for the transmission of PRRSV to uninfected pigs [29]. Seventeen point eight percent (79/444) of the asymptomatic pigs were positive in this study. Similar findings (15.02%, 70/466) were reported in a study that employed traditional RT-PCR in China [29]. However, this is first study to report the viral load using serum samples in asymptomatic PRRSV-infected pigs using ZNA probe-based real-time PCR. Four asymptomatic PRRSV-infected pigs were detected at levels as high as 4 log_10_ viral copies/μl (Figure 2). This result may be explained by differences in the susceptibility to PRRSV infection.

The present study also reinforces a the previous suggestion that PRRSV is one of the major viral agent for PRDC [30,31]. PRRSV viraemia was detected in 84.2% (112/133) of the PRDC pigs, ranging from 1.69 to 7.05 log_10_ PRRSV genome/μl (mean 4.28 ± 1.31 log_10_, median 4.21 log_10_). In contrast to the symptomatic pigs, the viraemia of the asymptomatic pigs ranged from 1.32 to 4.7 log_10_ PRRSV genome/μl (mean 2.72 ± 0.67 log_10_, median 2.75 log_10_). Moreover, based on the present results, it can be concluded that when pigs are infected with PRRSV, the amount of PRRSV in serum samples is significantly higher in PRDC pigs. Similar reports had been made for another major viral agent of PRDC, PCV2 [32-34]. However, there were no previous studies performed for this disease. The results obtained in the present study indicated that the PRRSV load in serum might be used to assess the importance of PRRS infection in a symptomatic case of PRDC.

## Conclusions

ZNA probe-based real-time PCR can be a useful tool to diagnose asymptomatic PRRSV-infected pigs. The presence of high PRRSV loads in a sample of animals (>10^4.2^ PRRSV genomes/μl of serum) is correlated to the presence of PRDC in pigs.

## Methods

### Animal, specimen collection and sample preparation

Serum samples were collected from 577 pigs from middle and southern Taiwan from 2012 to 2013. These animals included 444 healthy pigs and 133 symptomatic pigs (aged from 5 to 12 weeks old) that showed a clinical history of PRDC, such as listlessness, emaciation, hyperpnea or dyspnea. RNA extraction and reverse transcription were performed according to the procedures outlined in Chomczynski [35] and Lin [36], respectively. This study protocol was approved by the Animal Care and Use Committee of the National Pingtung University of Science and Technology.

### Primer and probe design

A conserved region of the M gene (the ORF 6 product) was identified in 125 nucleotide sequences from Asian (n = 74), European (n = 41), and American (n = 10) available from GenBank, which were aligned using the CLUSTAL W method and the MegAlign program (DNASTAR, Madison, WI). A 177-bp region was amplified from the ORF 6 gene of PRRSV using the primer pair PRRSV-M177F &-M177R (Table 5).

**Table 5:** Primers and ZNA probe used in the multiplex real-time PCR assay for the M gene

**Primer or probe**	**Sequence (5′-3′)**	**Primer length (bp)**	**Amplicon length (bp)**	**Position**^**a**^
PRRSV-M177F	CATTCTGGCCCCTGCCCA	18	177	14698-14715
PRRSV-M177R	ACCACTCCYYGYTTDACAGCT	21		14874-14854
NA Probe	FAM-CTCGTGTTGGGTGGCAGA-ZNA4 BHQ1	18		14834-14851

^a^Nucleotide numbering is based on the PRRSV isolate of strain VR-2332 (accession no. DQ176021).

### Construction of the plasmid DNA standard curves

A DNA fragment of the ORF 6 gene was amplified from the PRRSV vaccine (Ingelvac® PRRS MLV, Boehringer Ingelheim) using conventional PCR. The PCR products were cloned using the T&A cloning kit (Yeastern Biotech Co., Ltd., Taipei, Taiwan) and sequenced. The PRRSV plasmids were purified using a plasmid miniprep purification kit (GMbiolab Co., Ltd., Taichung, Taiwan) and quantified by measuring the OD260 using a spectrophotometer (Hitachi U2900, Dallas, TX, USA). A standard curve was generated using 10-fold dilutions (10^0^-10^6^ copies/μl) of the standard plasmid DNA.

### ZNA probe-based real-time PCR to detect PRRSV

The ZNA probe-based real-time PCR assays were performed using the LightCycler Nano (Roche Diagnostics, Mannheim, Germany). Each 10 μl reaction mixture contained 0.2 μM concentrations of the forward and reverse primers and 3 μl of the cDNA. The thermocycling conditions consisted of 10 min at 95°C and 45 cycles of 10 sec at 95°C, 10 sec at 55°C and 15 sec at 72°C. Each run included serial 10-fold dilutions of the standard plasmid DNA as a positive control and to construction the standard curve. A negative control that was missing the DNA template was included to detect any cross-contamination.

### Reproducibility and specificity of ZNA probe-based real-time PCR

The intra- (within-run) and inter- (between runs) assay reproducibility were evaluated using 10-fold serial dilutions of the standard plasmid DNA (from 10^1^-10^5^ copies per reaction), tested in triplicate on three different days. The coefficients of variation of the absolute copy number obtained from each dilution were calculated. The reproducibility of the method was also evaluated by repeatedly testing the clinical samples, as previously described [37]. The specificity of the ZNA probe-based real-time PCR assay was assessed by testing nucleic acid extracts of porcine classical swine fever, porcine circovirus type 2, porcine parvovirus and porcine pseudorabies virus.

### Statistical analysis

Student’s *t*-test was used to compare the viral loads between the various clinical symptom groups. The correlation between PRRSV and PRDC was evaluated using the chi-square test with Yate’s correction. *P* values <0.01 and <0.001 were considered significant and highly significant, respectively.

## Abbreviations

PRRS: Porcine reproductive and respiratory syndrome; PRRSV: Porcine reproductive and respiratory syndrome virus; PRDC: Porcine respiratory disease complex; RT-PCR: Reverse transcription polymerase chain reaction; PCVAD: Porcine circovirus associated disease; ZNA: Zip nucleic acids; Cq: Quantification cycles.

## Competing interests

The authors declare that they have no competing interests.

## Authors’ contributions

CNL designed the ZNA probe, analyzed the experiments data and wrote the manuscript. WHL participated sample collection and performed the ZNA probe-based real-time PCR. LNH contributed to the RNA extraction and reverse transcription. SYW performed serum sample separation and arranged the data for statistical analysis. MTC managed the study, provided materials and reagents, contributed to the interpretation of the data and co-wrote the manuscript. All of the authors read and approved the final manuscript.
